# Unveiling publishing patterns in the European Society of Endocrine Surgeons congress abstracts: a retrospective multicentric publication analysis

**DOI:** 10.1007/s13304-025-02216-y

**Published:** 2025-09-24

**Authors:** Max Schneider, Agata Dukaczewska, Dirk-Jan van Beek, Klaas Van Den Heede, Gunjan Sharma, Martin Almquist

**Affiliations:** 1 Department of Surgery, Klinik Landstraße, Wiener Gesundheitsverbund, Vienna, Austria; 2 Department of Surgery, Campus Charité Mitte and Campus Virchow-Klinikum, Charité–Universitätsmedizin Berlin, Freie Universität Berlin, Humboldt-Universität zu Berlin and Berlin Institute of Health, Charitéplatz 1, 10117 Berlin, Germany; 3 Department of Endocrine Surgical Oncology, University Medical Center Utrecht, Utrecht, Netherlands; 4 Department of General and Endocrine Surgery, Onze-Lieve-Vrouw (OLV) Hospital Aalst-Asse-Ninove, Aalst, Belgium; 5 Department of Surgery, Lund University Hospital, Lund, Sweden

**Keywords:** European Society of Endocrine Surgeons, Congresses, Abstracts, Publication rates, Research

## Abstract

Since 2004, the European Society of Endocrine Surgeons (ESES) has hosted biennial congresses as a platform for presenting key research. However, the publication rate of abstracts presented at these congresses is unknown. We searched for full publications using titles and authors of abstracts presented orally or as posters at ESES congresses from 2004 to 2018. Abstract factors, such as type of study and number of patients, were investigated in relation to publication rate, journal impact factor, and citation rates of published studies. Out of 733 abstracts presented at ESES during this period, 207 (28%) were presented orally and 528 (72%) as a poster, Overall, 456 of the abstracts (62%) were published, with a median time of 11.4 months from the ESES congress to publication [range − 64.5 to 156]. The median number of citations per published abstract was 21 [range 0–821], the average annual citation rate was 2.3 [0–54], and the median journal impact factor was 2.2 [0–21.3]. The median number of patients included per study was small, accounting for 71.5 [range 1–22,580]. While factors predicting higher chances for publication were oral format (OR 5), 2006 and 2008 ESES congresses (OR 21 and 12.6, respectively), larger sample sizes, oral presentation format, native English authorship, and randomized trials had higher annual citation rates. While the publication rates of ESES congress abstracts are high, collaborative efforts to conduct multicentric prospective studies could increase the publication rate of abstracts and enhance the overall scientific impact of the ESES congresses.

**Supplementary Information:**

The online version contains supplementary material available at 10.1007/s13304-025-02216-y.

## Introduction

The European Society of Endocrine Surgeons (ESES) has been convening biennial congresses since 2004, alternating with biennial meetings–workshops, uniting experts and practitioners across European locations. These gatherings serve as important venues for sharing and discussing new insights in the field, facilitated through oral and poster presentations. From its inception, the ESES was associated with Langenbeck’s Archives of Surgery as a journal for publishing research presented at the biennial congresses. Since 2020, this role has been taken over by the British Journal of Surgery.

In the initial stages of the scientific process, from conception to practice transformation, gatherings such as meetings and congresses play pivotal roles. These platforms offer an informed audience, conducive to deliberating on the feasibility, significance, and limitation of ideas. Empowered by this feedback, researchers can tailor their approaches, forge collaborations, and address challenges.

For the ESES society, maintaining a high standard of scientific discourse is imperative, both to attract participants and to enhance rapid dissemination of research findings among experts in the field to enable early application of research findings in daily clinical practice to advance patient care. To ensure high quality of the meetings, the ESES used an internal abstract selection process by reviewing members. Additionally, the selection process aimed to ensure a balanced geographical distribution across Europe [1].

Publication acceptance rates are serving as a commonly employed congress quality indicator [2, 3] and range from 29.2 to 43.0% [4, 5] in surgical specialties. Nevertheless, ensuring consistency between presentations and subsequent publications is vital for validating the integrity of both. Besides publication acceptance rates, journal impact factors (IF) and citation rates of published works are commonly utilized metrics for quantifying significance [6]. Additionally, factors such as patient volume, study design, and institutional affiliations of authors are recognized as potential factors influencing publication outcomes [7].

Little is known on publication patterns of studies presented at ESES congresses. Insights in publication acceptance rates and the quality of the published abstracts provide useful information for ESES steering committees and for researchers willing to submit their studies. In addition, factors associated with published abstracts and high-impact abstracts could be used to improve the abstract selection process and to design future studies in endocrine surgery. Therefore, this study aimed to assess the publication acceptance rates, the quality of accepted abstracts and factors associated with publications, and the quality of the publications of abstracts presented at ESES congresses.

## Methods

### Study design

**Fig. 1:**
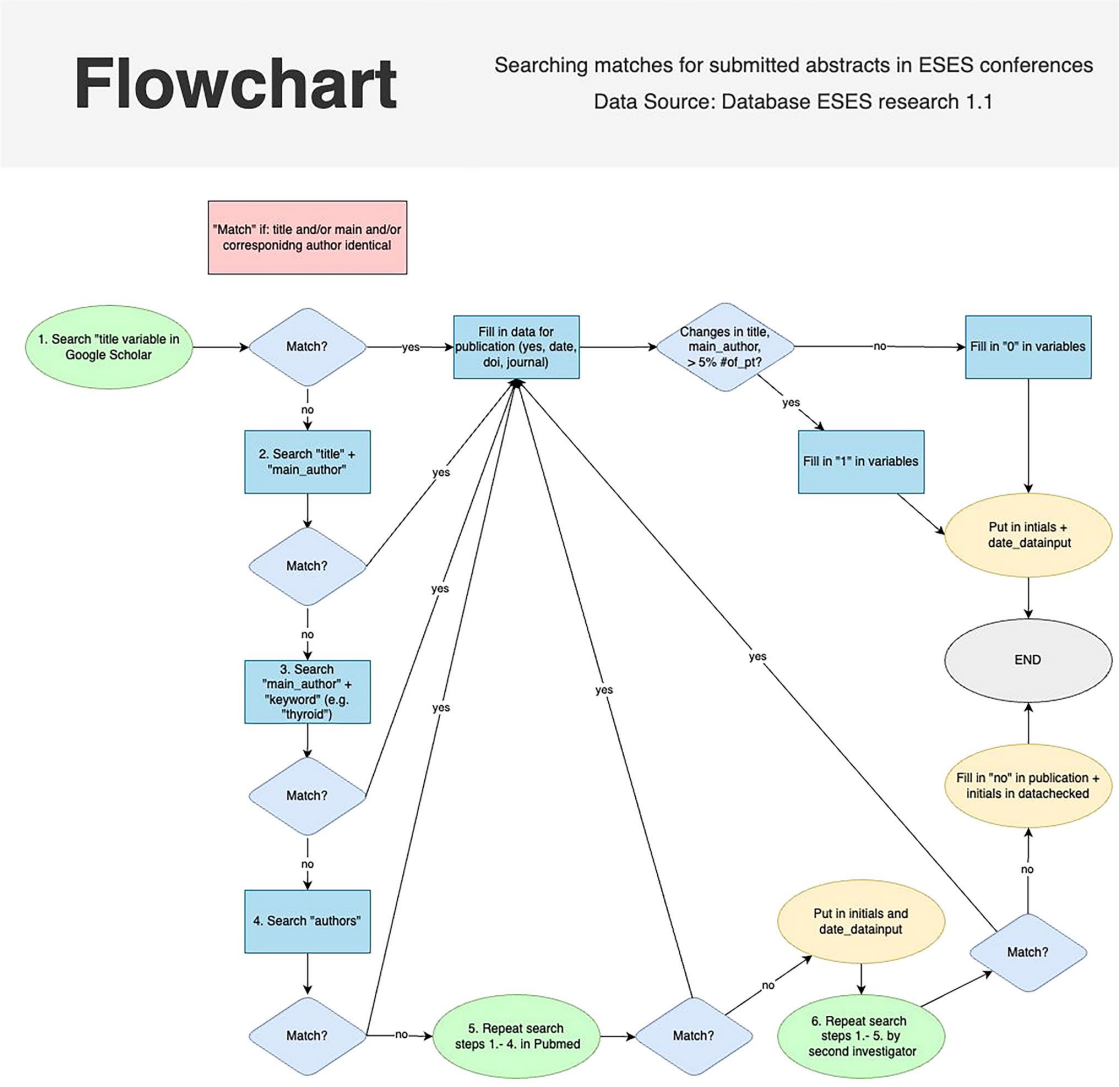
Flowchart presenting the search strategy for the full publication of congress abstracts

First, all abstracts presented at biennial European Society of Endocrine Surgeons (ESES) congresses (Pisa 2004, Krakow 2006, Barcelona 2008, Vienna 2010, Gothenburg 2012, Cardiff 2014, and Amsterdam 2018) were identified from congress abstract publications in Langenbeck’s Archives of Surgery [8–14]. Second, publications in journals belonging to the original ESES abstracts were searched for. The search for full publication of abstracts was conducted independently by four authors (AD, GS, KH and MS) in Pubmed or/and Google Scholar, as presented in the flowchart (Fig. 1). Both publications in non-indexed journals and abstracts published before the congress were included in the study. Potential changes between abstracts and final publications were assessed. If changes in authorship, title, or number of included patients or subjects were noted between the congress abstract and final publication, one point was given per change to quantify the differences.

### Data collection

We collected data on abstract titles, author names, last authors’ institutional affiliations, study type, research type, data collection methods, study field, and the number of included patients or subjects.

For published articles, title, author names, publication date, journal name, and number of patients or subjects included were recorded. Furthermore, journal speciality and journal impact factor (IF) in the year of the publication were documented for all journals. Abstracts published before their presentation at the congress and those published in non-indexed journals were included in the study. In addition, the number of citations were collected for all published papers. The searches for citation rates were conducted in Google Scholar during 3 weeks in October 2023.

To investigate the number of published abstracts by city and population, the number of abstracts published during the study period was gathered for each city and country of interest. To assess publication rates relative to population size, the total number of abstracts published was divided by the respective national population of each country.

### Outcome measures and definitions

The primary outcome was the percentage of published studies. Secondary outcomes included factors associated with a publication, the total citation number, the average annual citation rate calculated as total citations divided by the time between date of publication and date of citation capture (October 2023), and the IFs of the journals in which abstracts were published. In addition, factors associated with each of these outcomes were analysed. To analyse factors associated with a ‘high’ average annual citation rate and high IF, ‘high’ was defined as the top 25%.

### Statistical analysis

Continuous variables are presented as mean [standard deviation] or median [range] depending on the distribution. Categorical variables are presented as counts (percentages). Differences in continuous variables were assessed using Mann–Whitney *U* or Kruskal–Wallis tests, and categorical data were compared by using the Chi-square or Fisher’s exact test. Characteristics and outcomes were compared between the different ESES congresses.

Abstract characteristics between published and non-published abstracts were compared. The potential association between these characteristics and whether an abstract was published in a medical journal were assessed using univariable logistic regression providing odds ratios (OR) with corresponding 95% confidence intervals (CI). Characteristics evaluated for their association with an abstract being published were the year of ESES congress, number of patients in the presented abstract, subspeciality (thyroid, parathyroid, adrenal, neuroendocrine tumors (NET), or other), type of presentation (oral vs poster), type of research (clinical, basic/translational vs other), type of data collection (prospective, retrospective vs other), study design (randomized control trial (RCT), cohort or case control study, case report vs other), and language (native English speaking or non-native English speaking). UK, USA, India, Singapore, and Canada were considered as native English speaking.

In addition, we evaluated for the published abstracts whether the above-mentioned characteristics were associated with an annual citation rate, a total number of citations, and a journal IF above the 75th percentile.

The time from each ESES congress and the date of publication, either online or in-press, was assessed using Kaplan–Meier analysis. Kaplan–Meier curves were plotted, and estimated probabilities of being published were obtained [15]. Follow-up time started at the date of the ESES congress and ended at the date of (1) publication of the abstract or (2) last follow-up (i.e., July 1 st, 2023). The log-rank test was used for univariable Kaplan–Meier curve comparison.

Two-tailed *p* values < 0.05 were considered statistically significant. Data were analysed using SPSS version 26.0 (IBM Corp, New York, USA).

The heatmap presenting the publication centres in Europe was created using Tableau Desktop (Salesforce Inc., San Francisco, USA). World Bank database was used for population data (under CC BY 4.0 license).

## Results

Overall, some 733 abstracts were presented during the seven meetings. Of these, 207 (28.2%) were presented as oral presentation and 526 (71.8%) as poster presentation. No abstracts presented as posters were available for the Cardiff congress, while only 12 out of 142 abstracts for poster presentations were available for the Barcelona congress. Table 1 shows the overall characteristics. The median number of patients per study was 71.5 [1-22580]. Most abstracts represented clinical studies and cohort studies with retrospective data collection. Over half of the abstracts (53.6%) focussed on thyroid disorders/surgery, followed by parathyroid disorders/surgery (23.5%), and adrenal disorders/surgery (13.6%).

**Table 1:** Baseline characteristic of abstracts presented at the ESES congresses from 2004 to 2018

Variable	*N* = 733 (%)
Median [range] number of patients per abstract, *n* = 694 (94.7%)	71.5 [1–22580]
Subspeciality	
Thyroid	393 (53.6%)
Parathyroid	172 (23.5%)
Adrenal	100 (13.6%)
NET	49 (6.7%)
Study type	
Original	729 (99.4%)
Literature	4 (0.5%)
Type of research	
Clinical	653 (89.0%)
Basic/translational	76 (10.4%)
Data collection	
Prospective	225 (30.7%)
Retrospective	471 (64.3%)
English language	
Native	70 (9.5%)
Non-native	663 (90.5%)
Study design	
RCT	21 (2.9%)
Cohort	512 (69.8%)
Case–control	57 (7.8%)
Case report	59 (8.0%)
Type of presentation	
Oral	207 (28.2%)
Poster	526 (71.8%)
Publication	456 (62.2%)
Median [range] time between presentation and publication in months, *n* = 455	11.4 [− 64.5 to 156]
Median [range] total citation number, *n* = 449	21 [0–821]
Median [range] citation rate per year, *n* = 446	2.3 [0–54]
Median [range] journal impact factor, *n* = 452	2.2 [0–21.3]

*NET* neuroendocrine tumours, *RCT* randomized controlled trial

### Changes over time

**Fig. 2:**
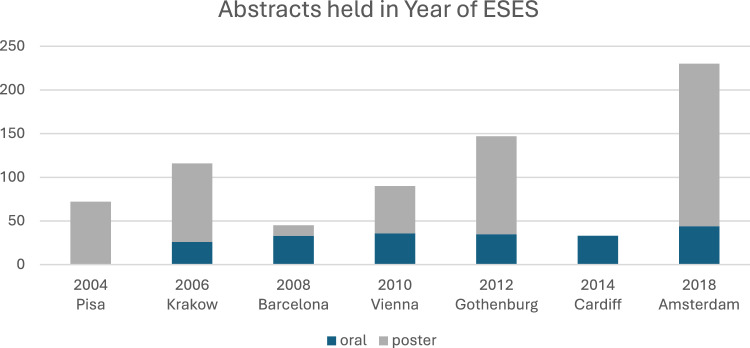
Number of accepted abstracts in the ESES congresses according to the type and year of presentation

Seven ESES congresses were analysed. The number of presented studies, for which abstracts were available retrospectively, ranged from 33 to 230 per congress (Fig. 2). No clear trends were observed over the years (Supplementary Table 1). The percentage of published abstracts ranged from 31.9 to 98.9%, respectively. For ESES 2004, having the longest follow-up time, the percentage was 31.9%, whereas for ESES 2018, with the shortest follow-up time, the percentage was 46.5%. Regarding subspecialities, type of research, and the number of patients, no relevant differences were seen between the different congresses. The percentage of oral presentations significantly differed between the years ranging from 0 to 100%, *p* < 0.001. Prospective data collection ranged from 18.2 to 35.6%. For all congresses, most studies were cohort studies (approximately 70%). In 2008, relatively more randomized controlled trials (RCT’s) were found (17.8%). As expected, published studies from the earliest congresses had the highest number of total citations, but the average annual number of citations was similar over the years.

### Publication

Overall, 456 of 733 abstracts (62.2%) were published. The median time from the ESES congress until the date of publication was 11.4 months [range − 64.5 to 156]. Sixty-three studies were linked to abstracts being published before the ESES congress, of which 36 were published within 12 months before the ESES congress.

After 12, 24, 36, 48, and 60 months post-congress, 25.1%, 38%, 45.6%, 49.6%, and 52.9% abstracts were published, respectively.

Changes in the title were observed in almost half of the abstracts (45.6%), changes in authors in one-quarter (24.1%), and changes in the number of patients in one-third (36.9%). The overall change score could be computed for 452 publishes abstracts (99.1%) and a score of ‘0’ was most frequently observed (43.4%). Major changes, meaning a score of 3, was reported for 38 studies (8.4%).

The median total number of cites per abstracts was 21 [range 0–821], the median average annual citation rate 2.3 [0–54], and the median journal impact factor (IF) 2.2 [0–21.3].

Nineteen journals published four or more congress abstracts (Supplementary Table 2). Of these 19 journals, the vast majority (*n* = 15) were surgical journals. All but two of these were general surgical journals. Most abstracts were published in Langenbeck’s Archives of Surgery (*n* = 95, 20.8% of published abstracts), World Journal of Surgery (*n* = 32, 7.0%), Surgery (*n* = 12, 2.9%), and the British Journal of Surgery (*n* = 12, 2.9%).

### Factors associated with a publication

Table 2 reports differences between published and unpublished abstracts. Published abstracts were more often presented as oral presentation (39.7% versus 12.3%, *p* < 0.001), had more prospective data collection (33.8% versus 25.8%, *p* < 0.001), and were less often case reports (5.5% versus 12.3%, *p* < 0.001) compared with unpublished abstracts. Corresponding odds ratios (ORs) are listed in Table 2. No differences were seen between subspecialities. In multivariable logistic regression, ESES 2006 (OR 21.0 [95% CI 8.5–51.6]), ESES 2010 (OR 125.9 [14.9–1056], oral presentations (OR 5.4 [3.1–9.6]), and case reports (OR 0.2 [0.003–0.9]) served as protective factors or publication after adjusting for subspeciality, type of research, and method of data collection.

**Table 2:** Factors associated with publication of abstracts presented at ESES congresses in a medical journal

Variable	Publication		*p* value	Univariable analysis		Multivariable analysis	
	Yes (*N* = 456)	No (*N* = 277)		OR	95% CI	OR	95% CI
Year							
2004	23 (5.0%)	49 (17.7%)	< 0.001	Ref	–	Ref	–
2006	108 (23.7%)	8 (2.9%)		28.8	12.0–68.8	21.0	8.5–51.6
2008	33 (7.2%)	12 (4.3%)		5.9	2.6–13.4	1.4	0.5–3.8
2010	89 (19.5%)	1 (0.4%)		189.6	24.8–1446.9	125.9	15.0–1057
2012	75 (16.4%)	72 (26.0%)		2.2	1.2–4.0	1.4	0.8–2.7
2014	21 (4.6%)	12 (4.3%)		3.7	1.6–8.9	0.6	0.2–1.9
2018	107 (23.5%)	123 (44.4%)		1.9	1.1–3.2	1.2	0.7–2.2
Median [range] number of patients per abstract	71 [1–22580]	72 [1–15127]	0.539	–	–	–	–
Subspeciality							
Thyroid	242 (53.1%)	151 (54.4%)	0.623	1.0	0.6–1.5	1.4	0.7–2.5
Parathyroid	110 (24.1%)	62 (22.4%)		1.1	0.7–1.8	1.1	0.6–2.0
Adrenal	62 (13.6%)	38 (13.7%)		Ref	–	Ref	–
NET	33 (7.2%)	16 (5.8%)		1.3	0.6–2.6	1.0	0.4–2.5
Other	9 (2.0%)	10 (3.6%)		0.6	0.2–1.5	0.6	0.2–2.1
Type of presentation							
Oral	173 (37.9%)	34 (12.3%)	< 0.001	4.4	2.9–6.6	5.5	3.1–9.7
Poster	283 (62.1%)	243 (87.7%)		Ref	–	Ref	–
Type of research							
Clinical	404 (88.6%)	249 (89.9%)	0.705	1.9	0.3–14.4	12.3	0.2–705.6
Basic	50 (11.0%)	26 (9.4%)		1.6	0.3–11.6	35.1	0.5–2709
Other	2 (0.4%)	2 (0.7%)		Ref	–	Ref	–
Data collection							
Retrospective	269 (59.0%)	202 (72.9%)	< 0.001	Ref	–	Ref	–
Prospective	154 (33.8%)	71 (25.6%)		1.6	1.2–2.3	0.98	0.6–1.5
Not applicable	33 (7.2%)	4 (1.4%)		6.2	2.2–17.8	0.8	0.2–3.6
English language							
Native	38 (8.3%)	32 (11.6%)	0.150	Ref	–	Ref	–
Non-native	418 (91.7%)	245 (88.4%)		1.4	0.9–2.4	1.3	0.7–2.4
Study design							
RCT	19 (4.2%)	2 (0.7%)	< 0.001	4.0	0.9–18.6	0.5	0.05–5.1
Cohort	311 (68.2%)	201 (72.6%)		0.7	0.4–1.1	0.2	0.04–1.0
Case–control	42 (9.2%)	15 (5.4%)		1.2	0.6–2.5	0.3	0.06–1.9
Case report	25 (5.5%)	34 (12.3%)		0.3	0.2–0.6	0.2	0.03–0.9
Other including MA and SR	59 (12.9%)	25 (9.0%)		Ref	–	Ref	–

*MA* meta-analysis, *SR* systematic review

**Fig. 3:**
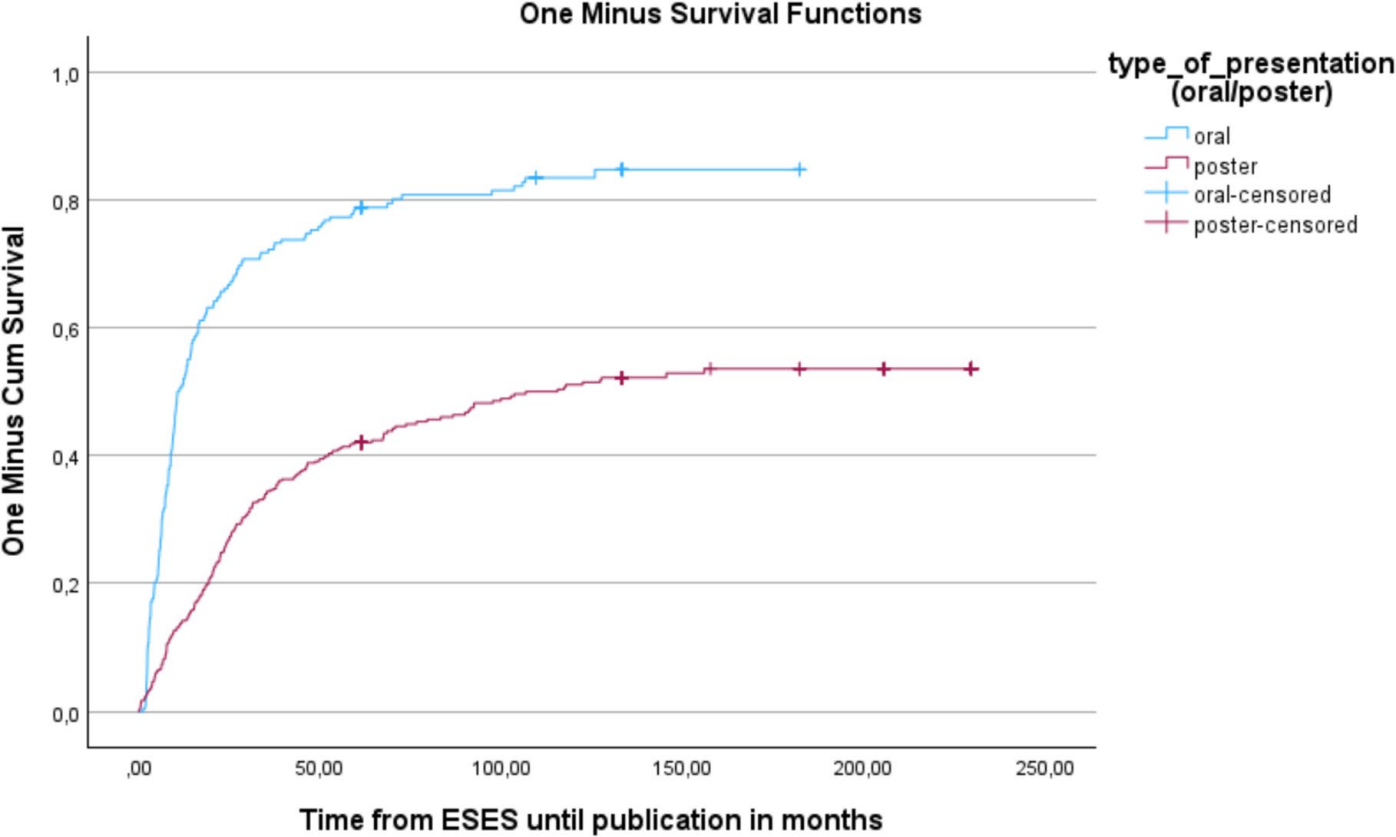
Kaplan–Meier analysis of time until publication for oral and poster presentations

Abstracts which were orally presented were published faster (median time from ESES congress to publication 10.6 months versus 106.7 months, log-rank *p* value < 0.001) (Fig. 3). The median times were 13.4 months for RCTs, 23.6 months for other studies including systematic reviews and meta-analyses, 28.6 months for case–control studies, 55.9 months for cohort studies, and not reached for case reports (log-rank *p* value < 0.001), respectively.

**Fig. 4:**
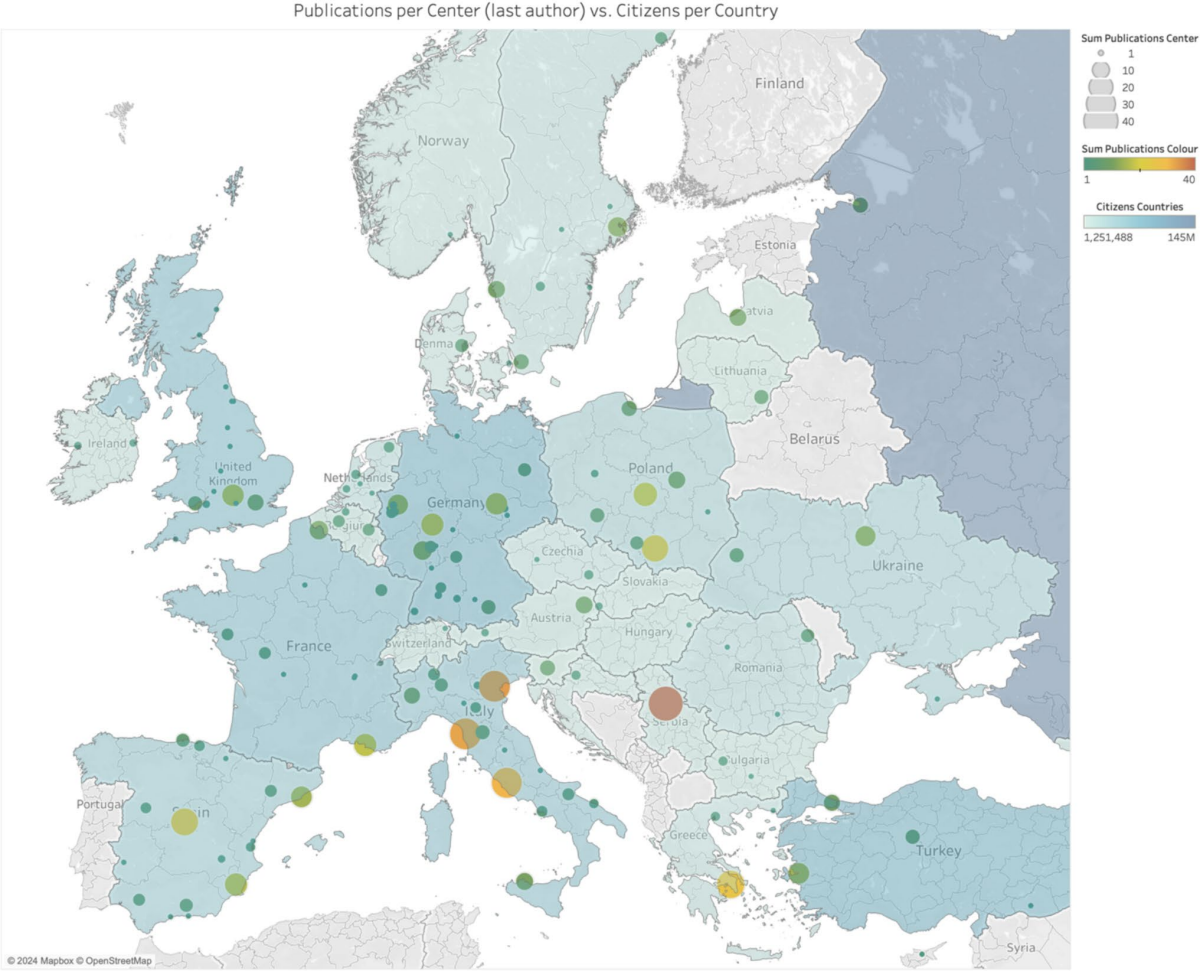
Heatmap of publication centres and population

As shown in Fig. 4, Belgrade, Padua, and Pisa had the highest number of published abstracts during the investigated time (39, 34 and 33, respectively). In relation to country’s population Serbia, Latvia, and Sweden showed the highest numbers of published abstracts (5.92, 5.84, and 4.00/per million citizens, respectively).

### Factors associated with a high annual citation rate, total citation number, and IF

In the published abstracts, factors associated with a high average annual citation rate are shown in Supplementary Table 3. For seven studies, no citation number could be found. Abstracts with a high average annual citation rate were oral presentations (53.6% versus 33.2%, *p* < 0.0001), had prospective data collection (42.9% versus 31.2%, *p* = 0.051), were RCTs (8.9% versus 2.7%, *p* = 0.003), issued from English native countries (13.4% versus 6.8%, *p* = 0.031), and had a higher median number of patients (95 versus 66 patients, *p* = 0.006) as compared to abstracts without a high average annual citation rate. In multivariable logistic regression, oral presentations had a high average annual citation rate (OR 2.2 [95% CI 1.3–3.7]) as compared to poster presentations, and papers from non-native English countries had a lower average annual citation rate compared to native countries (OR 0.4 [95% CI 0.2–0.8]), when adjusted for year of the congress, subspeciality, type of research, method of data collection, and study design.

Supplementary Table 4 presents the factors associated with a high journal IF at the time of publication. For 31 abstracts, the IF was unknown. In multivariable analysis, ESES 2010 (OR 0.2 [95% CI 0.1–0.8]) and abstracts from non-native English-speaking countries (OR 0.7 [95% CI 0.3–1.6]) were negatively associated with a high journal IF.

The most cited papers regarding the average annual number of citations are reported in Table 3.

**Table 3:** Publications of abstracts presented at ESES congresses with the highest annual citation rate

#	Abstract title	ESES congress year	Publication title	Journal	First and last author	DOI	Publication year	Average yearly cites
1	Complications to thyroid surgery: results as reported in a database from a multicenter audit comprising 3660 patients	2008	Complications to thyroid surgery: results as reported in a database from a multicenter audit comprising 3,660 patients	Langenbecks Arch Surg	Bergenfelz A, Lausen I	10.1007/s00423-008–0366-7	2008	53.99
2	Visualisation versus intraoperative neuromonitoring of the recurrent laryngeal nerves in thyroid surgery—a prospective randomized study	2008	Randomized clinical trial of visualization versus neuromonitoring of recurrent laryngeal nerves during thyroidectomy	Br J Surg	Barczyński A, Cichoń S	10.1002/bjs.6417	2009	35.72
3	Parathyroid in situ preservation to prevent hypocalcaemia after total thyroidectomy	2014	Importance of in situ preservation of parathyroid glands during total thyroidectomy	Br J Surg	Lorente-Poch L, Sitges-Serra A	10.1002/bjs.9676	2015	30.36
4	The SIPH study: surgery vs. medical observation in mild, asymptomatic pHPT. Preliminary results from a prospective randomised study	2006	Medical observation, compared with parathyroidectomy, for asymptomatic primary hyperparathyroidism: a prospective, randomized trial	Journal of Clinical Endocrinology & Metabolism	Bollerslev J, Rosen T	10.1210/jc.2006-1836	2007	24.92
5	Identification of cancer initiating cells in human thyroid cancer	2010	Tumorigenic and metastatic activity of human thyroid cancer stem cells	Cancer Research	Todaro M, Stassi G	10.1158/0008-5472.CAN-10–1994	2010	20.24

## Discussion

Our study demonstrated that the publication rate for abstracts presented at the European Society of Endocrine Surgeons (ESES) congresses is 62.2%. This figure notably exceeds the average. In comparison, the latest Cochrane review of reports of 425 scientific meetings showed a publication rate of 37.3% [7]. The publication rate of abstracts presented at surgical and endocrinology congresses accounted for 39.2% and 31.7%, respectively [7]. To our knowledge, no evaluation of publication rates of abstracts presented at endocrine surgical congresses have been conducted to date. However, it was shown that members of American Association of Endocrine Surgeons (AAES) surpassed other academic surgical faculty from the 50 highest NIH-funded universities and 5 prominent hospital-based surgical departments in the USA in publications and citations [16]. Consistent with these findings, our results reflect a significant interest of endocrine surgeons to disseminate research through publication as the most effective means to spread up-to-date knowledge and advance clinical practice.

Our findings align with previous studies, showing that oral presentations are more likely to be published than posters [7, 17–19]. Additionally, oral presentations are often published earlier. The same trend applies to studies based on prospective data collection [7]. Furthermore, our study revealed small patient cohorts (median of 71.5), a phenomenon likely attributed to the rarity of many endocrine diseases. Consistent with prior results, the small sample sizes did not correlate with a lower publication rate [7]. However, they were associated with lower annual citation rates. It has previously been shown that studies conducted in multiple centres or those involving clinics from various countries, and thus, including potentially larger patient cohorts, demonstrated higher publication rates [7, 20, 21]. Therefore, we propose encouraging collaboration among endocrine surgeons within the ESES network to achieve higher publication rates.

It has previously been reported that abstracts from native English-speaking countries are more frequently published than those from non-native English-speaking countries [17]. However, this trend was not reflected in our results, as the difference was not statistically significant. Thus, English proficiency is widespread among surgeons presenting at ESES congresses and does not appear to hinder publication. Nevertheless, manuscripts published by surgeons from native English-speaking countries had higher IFs and annual citation rates. Authors from non-native English-speaking countries may not always target high-impact journals due to various reasons such as perceived competition or preference for regional or national journals. Interestingly, the five publications with the highest annual citation rates came from authors from non-native English-speaking countries: Sweden, Poland, Spain, Norway, and Italy. Therefore, reaching a high scientific impact is feasible irrespective of the country from which the research originates.

The median journal impact factor (IF) of manuscripts analysed in the present study was 2.2. The reported IF for full publication of abstracts presented at surgical congresses ranged from 2.35 to 3.7 [5, 18–20, 22]. Thus, the IF of the published articles was lower than the average. This phenomenon may be attributed to a lower interest of editors of high-impact journals in endocrine diseases compared to other surgical specialties. Consequently, there is potential for improvement. Incorporating feedback on abstracts during presentations at ESES congresses could contribute to enhancing the quality of subsequent publications. Interestingly, the citation rates of articles had not been depicted in the two systematic reviews available to date analysing publication rates of abstracts initially presented at congresses [7, 17]. Citation rates represent scientific impact of particular articles, irrespective of IFs of journals they were published in. Our investigation shows a high total number of cites per abstract of 21, confirming a high scientific impact of the research presented at the ESES congresses.

Many abstracts presented at the congresses contain data of ongoing studies. This not only allows prompt sharing of new findings, but also provides the authors with the opportunity to gain additional perspectives through discussion with the scientific community and helps prepare the eventual publication [21]. Thus, discrepancies between the presented abstract and the final publication may occur. To quantify the discrepancies, Ataei et al. categorized changes into four grades. Grade 0 was assigned if there were no changes between the abstract and the manuscript, and grades 1, 2, and 3 were applied when manuscript contained additional data or investigations that did or did not lead to a different conclusion [23]. In our view, the score proposed by Ataei et al. allows for subjective interpretation of the disparities between the abstract and the final manuscript. Denadai et al. classified variations into major ones (changes in purpose, methods, study design, sample size, statistical analysis, results, and conclusions) and minor ones (modifications in the title and authorship) [24]. Studies in which the score was implemented showed major and minor variations in 65–76% and 76–96%, respectively, [21, 25–27] and most common changes were observed in authorship [21, 24]. In our opinion, modifying the study purpose, design, and/or methods of data collection disqualifies an assumption that a full article still corresponds to an abstract presented at a congress. Conversely, if a study is conducted over an extended period, changes in the number of patients included and an authorship may occur. These routine alterations in the study process do not change its fundamental nature. Therefore, we proposed a simplified scoring system, to assess title, authorship, and sample size changes. In our study, changes in the title were the most prevalent, followed by alterations in authorship. We observed changes in 56.6% of the full publications and changes in all three variables in only 8.4% of the studies. Consequently, the data presented at the ESES congresses closely align with the published data and can be considered reliable.

The time to publication averaged 11.4 months. Of note, 63 studies (13.8%) were published before the congress. Additionally, as 27 studies (5.9%) were published more than 12 months before the congress, this occurrence cannot be attributed solely to the time between abstract submission and the ESES meeting. It had been reported that up to 16.9% of abstract are published before the scientific meetings [28]. The intention of the authors to draw attention to the results of their studies via a presentation at a congress is understandable. However, presentations at a congress should ideally focus on the newest, unpublished findings. Therefore, a more rigorous selection process should be implemented for abstracts presented the ESES meetings.

Our data collection was carried out manually. Despite the effort of two independent researchers looking for a full publication of each abstract, manual data entry may still be prone to failures. So far, two studies had applied automated searches for full publications of congress abstracts, using a Python code [21] and a SAS software package [29]. Advances in automated data collection will likely soon facilitate the investigation of the entire congress abstract publication, providing participants with valuable feedback regarding the impact of scientific meetings.

Our study had limitations. First, we conducted a thorough analysis of all retrospectively available abstracts presented at the ESES congresses held at least five years before the current study. Consequently, full publications of abstracts published after this time frame, particularly those presented at the last congress included in this study (ESES congress in Amsterdam in 2018) could have been overlooked. However, a Cochrane review had shown, that most papers are published within 3–4 years after a scientific meeting [17]. Second, the limitations include some incomplete congress data. Since no abstracts presented as posters were available for the Cardiff congress, while only 12 out of 142 abstracts for poster presentations were available for the Barcelona congress, a bias in poster selection for our study could explain the rather high publication rates, as compared to previous publications, since a number of posters were not included in our study and the selection of abstracts is skewed towards oral presentations. Moreover, publications in less accessible or non-English journals could have been missed. However, this limitation was effectively addressed by using two comprehensive sources for the publication search (PubMed and Google Scholar) and having two independent researchers to double-check the searches. This process resulted in identifying an overall high publication rate. This thorough approach strengthens the credibility of our findings, suggesting that the limitations had minimal impact on the results. While the study was conducted retrospectively, it would be beneficial to have a prospectively maintained database that includes data from additional congresses. An analysis of a larger database would provide a more comprehensive understanding of the publication trends and outcomes.

## Conclusions

Our study revealed that the publication rates of ESES congress abstract exceed the average. The likelihood of publication was significantly higher for studies with prospective data collection and those presented orally. Even though the publication rate was not negatively associated with a small sample size, it could be amplified by fostering collaborative initiatives within ESES to incorporate a larger number of patients into prospective studies, according to previous results.

## Supplementary Information

Below is the link to the electronic supplementary material.

Supplementary file1 (DOCX 31 KB)

## Data Availability

Data is available on request.
