# Supplementary material for: Unveiling publishing patterns in the European Society of Endocrine Surgeons congress abstracts: a retrospective multicentric publication analysis
Source: Updates Surg. 2025 Sep 24;77(8):2205–16. doi: 10.1007/s13304-025-02216-y (PMC12630310; doi:10.1007/s13304-025-02216-y)
Supplement: Supplementary file 1 — Supplementary file1 (DOCX 31 KB) [file 13304_2025_2216_MOESM1_ESM.docx]

**Supplement**

Supplementary Table 1 – Characteristics of publication patterns of abstracts presented at ESES congresses by year

|  | **Year** | | | | | | |  |
| --- | --- | --- | --- | --- | --- | --- | --- | --- |
| **Variable** | **2004** | **2006** | **2008** | **2010** | **2012** | **2014** | **2018** |  |
| No. presented | 72 | 116 | 45 | 90 | 147 | 33 | 230 |  |
| No. published | 23 (31.9%) | 108 (93.1%) | 33 (73.3%) | 89 (98.9%) | 75 (51.0%) | 21 (63.6%) | 107 (46.5%) | P<0.001 |
| Subspecialty  Thyroid  Parathyroid  Adrenal  NET  Other/combined | 41 (56.9%)  16 (22.2%)  8 (11.1%)  3 (4.2%)  4 (5.6%) | 57 (49.1%)  22 (19.0%)  19 (16.4%)  15 (12.9%)  3 (2.6%) | 24 (53.3%)  12 (26.7%)  7 (15.6%)  1 (2.2%)  1 (2.2%) | 49 (54.4%)  24 (26.7%)  11 (12.2%)  6 (6.7%)  0 (0%) | 66 (44.9%)  42 (28.6%)  22 (15.0%)  13 (8.8%)  4 (2.7%) | 16 (48.5%)  7 (21.2%)  6 (18.2%)  2 (6.1%)  2 (6.1%) | 140 (60.9%)  49 (21.3%)  27 (11.7%)  9 (3.9%)  5 (2.2%) | P=0.172 |
| Type of presentation  Oral  Poster | 0 (0%)  72 (100%) | 26 (22.4%)  90 (77.6%) | 33 (73.3%)  12 (26.7%) | 36 (40.0%)  54 (60.0%) | 35 (23.8%)  112 (76.2%) | 33 (100%)  0 (0%) | 44 (19.1%)  186 (80.9%) | P<0.001 |
| Type of research  Clinical  Basic/translational  Other | 63 (87.5%)  7 (9.7%)  2 (2.8%) | 107 (92.3%)  9 (7.8%)  0 (0%) | 36 (80.0%)  9 (20.0%)  0 (0%) | 80 (88.9%)  8 (8.9%)  2 (2.2%) | 131 (89.1%)  16 (10.9%)  0 (0%) | 31 (93.9%)  2 (6.1%)  0 (0%) | 205 (89.1%)  25 (10.9%)  0 (0%) | P=0.055 |
| Data collection  Prospective  Retrospective  Not applicable | 17 (23.6%)  55 (76.4%)  0 (0%) | 31 (26.7%)  73 (62.9%)  12 (10.3%) | 16 (35.6%)  20 (44.4%)  9 (20.0%) | 29 (32.2%)  47 (52.2%)  14 (15.6%) | 56 (38.1%)  91 (61.9%)  0 (0%) | 6 (18.2%)  25 (75.8%)  2 (6.1%) | 70 (30.4%)  160 (69.6%)  0 (0%) | P<0.001 |
| English language  Native  Non native | 4 (5.6%)  68 (94.4%) | 2 (1.7%)  114 (98.3%) | 4 (8.9%)  41 (91.1%) | 7 (7.8%)  83 (92.2%) | 11 (7.5%)  136 (92.5%) | 9 (27.3%)  24 (72.7%) | 33 (14.3%)  197 (857%) | P<0.001 |
| Study design  RCT  Cohort  Case control  Case report  Systematic review  Meta-analysis  Survey  Other | 0 (0%)  59 (81.9%)  0 (0%)  6 (8.3%)  0 (0%)  0 (0%)  0 (0%)  7 (9.7%) | 4 (3.4%)  85 (73.3%)  10 (8.6%)  6 (5.2%)  0 (0%)  0 (0%)  2 (1.7%)  9 (7.8%) | 8 (17.8%)  20 (44.4%)  8 (17.8%)  0 (0%)  0 (0%)  0 (0%)  0 (0%)  9 (20.0%) | 3 (3.3%)  63 (70.0%)  9 (10.0%)  3 (3.3%)  0 (0%)  0 (0%)  0 (0%)  12 (13.3%) | 4 (2.7%)  100 (68.0%)  5 (3.4%)  22 (15.0%)  0 (0%)  14 (2.7%) (0.7%)  11 (7.5%) | 0 (0%)  25 (75.8%)  6 (18.2%)  0 (0%)  0 (0%)  0 (0%)  0 (0%)  2 (6.1%) | 2 (0.9%)  160 (69.6%)  19 (8.3%)  22 (9.6%)  1 (0.4%)  0 (0%)  5 (2.2%)  21 (9.1%) | P<0.001 |
| Median [range] No. of patients | 59 [1-2043] | 71 [1-20802] | 72 [6-14635] | 86.5 [1-3737] | 65.5 [1-15127] | 80.5 [5-987] | 77 [1-22580] | 0.249 |
| Median [range] time from congress to publication in months | 10.6 [-23.4-127.6] | 14.4 [-64.5-145.5] | 4.1 [-0.8-125.8] | 6.2 [-41.4-155.6] | 9.9 [-52.7-92.3] | 9.5 [5.9-68.5] | 16.3 [-52.6-59.5] | 0.178 |
| Number of citations | 54 [4-132] | 24.5 [0-405] | 47 [0-821] | 32.5[0-266] | 23 [0-157] | 20 [2-264] | 8 [0-115] | <0.001 |
| Average cites/year | 2.9 [0.3-7.8] | 1.7 [0-24.9] | 3.4 [0-54.0] | 2.8 [0-20.2] | 2.1 [0-14.3] | 2.9 [0.3-20.4] | 2.3 [0-19.5] | 0.032 |
| Impact factor | 2.1 [0.2-6.1] | 1.9 [0.0-10.2] | 1.9 [0.3-6.8] | 2.2 [0.0-21.3] | 2.3 [0.1-8.7] | 2.6 [0.2-5.7] | 2.4 [0.1-10.9] | 0.029 |

NET, neuroendocrine tumors; RCT, randomised controlled trial

Supplementary Table 2 – Journals with most published abstracts presented at ESES congresses

| **#** | **Journal title** | **N** | **% of total** | **% of published** | **Journal Specialty** | **Type of surgical journal** | **5-year IF*** |
| --- | --- | --- | --- | --- | --- | --- | --- |
| 1 | Langenbecks Archives of Surgery | 95 | 13.0% | 20.8% | Surgery | General surgery | 2.4 |
| 2 | World Journal of Surgery | 32 | 4.4% | 7.0% | Surgery | General surgery | 3.0 |
| 3 | SURGERY | 12 | 1.8% | 2.9% | Surgery | General surgery | 3.3 |
| 4 | British Journal of Surgery | 12 | 1.6% | 2.6% | Surgery | General surgery | 8.6 |
| 5 | The American Journal of Surgery | 10 | 1.4% | 2.2% | Surgery | General surgery | 2.5 |
| 6 | Acta Chirurgica Belgica | 10 | 1.4% | 2.2% | Surgery | General surgery | 0.8 |
| 7 | Endokrynologia Polska | 9 | 1.2% | 2.0% | Endocrinology | - | 1.8 |
| 8 | Annals of Surgery | 7 | 1.0% | 1.5% | Surgery | General surgery | 9.1 |
| 9 | Surgical Endoscopy | 7 | 1.0% | 1.5% | Surgery | Minimally invasive surgery | 3.1 |
| 10 | European Journal of Endocrinology | 6 | 0.8% | 1.3% | Endocrinology | - | 5.7 |
| 11 | Annals of Surgical Oncology | 5 | 0.7% | 1.1% | Surgery | Surgical Oncology | 4.0 |
| 12 | BJS Open | 5 | 0.7% | 1.1% | Surgery | General surgery | 3.9 |
| 13 | Head & Neck | 5 | 0.7% | 1.1% | ENT | - | 2.4 |
| 14 | Journal of Clinical Endocrinology & Metabolism | 5 | 0.7% | 1.1% | Endocrinology | - | 5.3 |
| 15 | [Cirugía Española](https://www.elsevier.es/es-revista-cirugia-espanola-36) | 4 | 0.5% | 0.9% | Surgery | General surgery | NA |
| 16 | Il Giornale di Chirurgia | 4 | 0.5% | 0.9% | Surgery | General surgery | 0.6 |
| 17 | International Journal of Surgery | 4 | 0.5% | 0.9% | Surgery | General surgery | 8.9 |
| 18 | JAMA Surgery | 4 | 0.5% | 0.9% | Surgery | General surgery | 16.3 |
| 19 | Journal of Surgical Research | 4 | 0.5% | 0.9% | Surgery | General surgery | 2.0 |

IF, impact factor; ENT, ear, nose and throat; NA, not available; *data retrieved in March, 2025

Supplementary Table 3 – Factors associated with high annual citation rate (above 75^th^ percentile) of abstracts presented at ESES congresses

| **Variable** | **Top 25% of average annual citation rate** | |  | **Univariable analysis** | | **Multivariable analysis** | |  |
| --- | --- | --- | --- | --- | --- | --- | --- | --- |
|  | Yes (N=112) | No (N=337) | p-value | OR | 95% CI | OR | 95% CI |  |
| Year  2004  2006  2008  2010  2012  2014  2018 | 6 (5.4%)  21 (18.8%)  14 (12.5%)  27 (24.1%)  13 (11.6%)  6 (5.4%)  25 (22.3%) | 17 (5.0%)  81 (24.0%)  19 (5.6%)  61 (18.1%)  62 (18.4%)  15 (4.5%)  82 (24.3%) | P=0.102 | Ref.  0.7  2.1  1.3  0.6  1.2  0.9 | -  0.3 – 2.1  0.7 – 6.7  0.4 – 3.5  0.2 – 1.8  0.3 – 4.3  0.3 – 2.4 | Ref.  0.5  0.8  0.8  0.3  0.4  0.5 | -  0.2 – 1.6  0.2 – 3.1  0.2 – 2.4  0.1 – 1.1  0.9 – 1.7  0.2 – 1.4 |  |
| Median [range] number of patients per abstract, n=426 | 95 [2-12485] | 66 [1-22580] | P=0.006 | - | - | - | - |  |
| Subspecialty  Thyroid  Parathyroid  Adrenal  NET  Other | 64 (57.1%)  23 (20.5%)  14 (12.5%)  9 (8.0%)  2 (1.8%) | 175 (51.9%)  87 (25.8%)  47 (13.9%)  21 (6.2%)  7 (2.1%) | P=0.743 | 1.2  0.9  Ref.  1.4  0.96 | 0.6 – 2.4  0.4 – 1.9  -  0.5 – 3.8  0.2 – 5.2 | 1.2  0.9  Ref.  1.8  1.2 | 0.6 – 2.6  0.4 – 2.1  -  0.6 – 5.1  0.2 – 7.7 |  |
| Type of presentation  Oral  Poster | 60 (53.6%)  52 (46.4%) | 112 (33.2%)  225 (66.8%) | P<0.001 | 2.3  Ref. | 1.5 – 3.6  - | 2.2  Ref. | 1.3 – 3.7  - |  |
| Type of research  Clinical  Basic  Other | 100 (89.3%)  11 (22.4%)  1 (0.9%) | 298 (88.4%)  38 (11.3%)  1 (0.3%) | P=0.657 | 2.9  0.3  Ref. | 0.02 – 5.0  0.02 – 5.4  - | 0.2  0.2  Ref. | - 1. – 4.1   0.01 – 5.5  - |  |
| Data collection  Retrospective  Prospective  Not applicable | 59 (52.7%)  48 (42.9%)  5 (4.5%) | 204 (60.5%)  105 (31.2%)  28 (8.3%) | P=0.051 | Ref.  1.6  0.6 | -  1.0 – 2.5  0.2 – 1.7 | Ref.  1.1  0.3 | -  0.7 – 1.9  0.1 – 1.3 |  |
| English language  Native  Non native | 15 (13.4%)  97 (86.6%) | 23 (6.8%)  314 (93.2%) | P=0.031 | Ref.  0.5 | -  0.2 – 0.9 | Ref  0.4 | -  0.2 – 0.8 | |
| Study design  RCT  Cohort  Case control  Case report  Other + MA + SR | 10 (8.9%)  73 (65.2%)  14 (12.5%)  1 (0.9%)  14 (12.5%) | 9 (2.7%)  233 (69.1%)  27 (8.0%)  24 (7.1%)  44 (13.1%) | P=0.003 | 3.5  0.99  1.6  0.1  Ref. | 1.2 – 10.3  0.5 – 1.9  0.7 – 3.9  0.02 – 1.06  - | 1.6  0.7  1.1  0.2  Ref. | 0.3 – 8.7  0.2 – 3.2  0.2 – 5.6  0.02 – 2.2  - |  |

NET, neuroendocrine tumors; RCT, randomized controlled trials; MA, metaanalysis; SR, systematic review

Supplementary Table 4 – Factors associated with high impact factor (above 75^th^ percentile) of abstracts presented at ESES congresses

| **Variable** | **Top 25% impact factor** | |  | **Univariable analysis** | | **Multivariable analysis** | |  |
| --- | --- | --- | --- | --- | --- | --- | --- | --- |
|  | Yes (N=109) | No (N=316) | p-value | OR | 95% CI | OR | 95% CI |  |
| Year  2004  2006  2008  2010  2012  2014  2018 | 8 (7.3%)  23 (21.1%)  12 (11.0%)  12 (11.0%)  15 (13.8%)  4 (3.7%)  35 (32.1%) | 13 (4.1%)  71 (22.5%)  21 (6.6%)  73 (23.1%)  55 (17.4%)  17 (5.4%)  66 (20.9%) | P=0.019 | Ref.  0.5  0.9  0.3  0.4  0.4  0.9 | -  0.2 – 1.4  0.3 – 2.9  0.1 – 0.8  0.2 – 1.3  0.1 – 1.6  0.3 – 2.3 | Ref.  0.4  0.9  0.3  0.4  0.5  0.9 | -  0.2 – 1.3  0.2 – 3.5  0.1 – 0.8  0.1 – 1.4  0.1 – 2.2  0.3 – 2.5 |  |
| Number of patients abstract (median [range]), n=426 | 55 [3-22580] | 80.5 [1-20802] | P=0.278 | - | - | - | - |  |
| Subspecialty  Thyroid  Parathyroid  Adrenal  NET  Other | 57 (52.3%)  22 (20.2%)  18 (16.5%)  9 (8.3%)  3 (2.8%) | 172 (54.4%)  81 (25.6%)  40 (12.7%)  18 (5.7%)  5 (1.6%) | P=0.498 | 0.7  0.6  Ref.  1.1  1.3 | 0.4 – 1.4  0.3 – 1.3  -  0.4 – 2.9  0.3 – 6.2 | 0.6  0.6  Ref.  1.3  1.2 | 0.3 – 1.3  0.3 – 1.4  -  0.5 – 3.7  0.2 – 6.4 |  |
| Type of presentation  Oral  Poster | 36 (33.0%)  73 (67.0%) | 129 (40.8%)  187 (59.2%) | P=0.150 | Ref.  0.7 | -  0.5 – 1.1 | Ref.  0.6 | -  0.3 – 1.0 |  |
| Type of research  Clinical  Basic  Other | 92 (84.4%)  31 (9.8%)  0 (0%) | 283 (89.6%)  31 (9.8%)  2 (0.6) | P=0.189 | NA | NA | NA | NA |  |
| Data collection  Retrospective  Prospective  Not applicable | 63 (57.8%)  39 (35.8%)  7 (6.4%) | 182 (57.6%)  109 (34.5%)  25 (7.9%) | P=0.870 | Ref.  1.0  0.8 | -  0.7 – 1.6  0.3 – 2.0 | Ref.  1.1  1.2 | -  0.6 – 2.0  0.3 – 4.8 |  |
| English language  Native  Non-native | 15 (13.4%)  97 (86.6%) | 23 (6.8%)  314 (93.2%) | P=0.031 | Ref.  0.8 | -  0.4 – 1.7 | Ref  0.7 | -  0.3 – 1.6 | |
| Study design  RCT  Cohort  Case control  Case report  Other + MA + SR | 6 (5.5%)  73 (67.0%)  12 (11.0%)  0 (0%)  18 (16.5%) | 13 (4.1%)  215 (68.0%)  27 (8.5%)  22 (7.0%)  39 (12.3%) | P=0.049 | 1.0  0.7  0.96  0.0  Ref. | 0.3 – 3.1  0.4 – 1.4  0.4 – 2.3  NA  - | 2.4  1.5  1.8  0.0  Ref. | 0.4 – 14.6  0.3 – 7.1  0.3 – 9.9  NA  - |  |

NET, neuroendocrine tumors; NA, not available; RCT, randomized controlled trial; MA, meta-analysis; SR, systematic review
